# Increased expression of tribbles homolog 3 predicts poor prognosis and correlates with tumor immunity in clear cell renal cell carcinoma: a bioinformatics study

**DOI:** 10.1080/21655979.2022.2086380

**Published:** 2022-06-20

**Authors:** Xin-Qiang Wu, Xi Tian, Fu-Jiang Xu, Yue Wang, Wen-Hao Xu, Jia-Qi Su, Yuan-Yuan Qu, Jian-Yuan Zhao, Hai-Liang Zhang, Ding-Wei Ye

**Affiliations:** a Department of Urology, Fudan University Shanghai Cancer Center, State Key Laboratory of Genetic Engineering, Collaborative Innovation Center for Genetics and Development, School of Life Sciences, Fudan University, Shanghai, China; b Department of Oncology, Shanghai Medical College, Fudan University, Shanghai, P.R. China; c Key Laboratory of Children’s Environmental Health, Xinhua Hospital, Shanghai Jiao Tong University School of MedicineInstitute for Developmental and Regenerative Cardiovascular Medicine, MOE-Shanghai, Shanghai, China

**Keywords:** TRIB3, ccRCC, biological significance, immune landscape characterization

## Abstract

Tribbles homolog 3 (TRIB3), a pseudokinase that regulates multiple intracellular signaling pathways, has been reported to promote the growth of multiple tumors. However, its role in clear cell renal cell carcinoma (ccRCC) remains unelucidated. We evaluated the role of TRIB3 in ccRCC using publicly available data from The Cancer Genome Atlas and analyzed its relationship with the tumor microenvironment; moreover, we used gene knockout and overexpression techniques to detect the effects of TRIB3 on the biological behavior of ccRCC cells. RT-qPCR and western blotting were used to detect transfection efficiency, and the invasiveness of ccRCC cells was determined by Transwell migration assays. We found that TRIB3 overexpression was significantly associated with increased grade, stage, and distant metastasis, positively correlated with ccRCC invasiveness, and also an independent risk factor for overall survival (OS). In addition, 361 differentially expressed genes (DEGs) related to TRIB3 were identified. Functional enrichment analysis showed that DEGs were mainly enriched in humoral immune responses, collagen-containing extracellular matrix, and serine hydrolase activity. Immune landscape characterization revealed that TRIB3 expression was significantly and negatively associated with CD8^+^ T and hematopoietic stem cells, whereas it was positively associated with NK T and macrophage M1 cells. Single-cell sequencing showed that localization and binding targets of TRIB3 mainly involved monocytes/macrophages and CD4^+^ and CD8^+^ T cells. Overall, our study revealed that elevated TRIB3 expression represents a promising prognostic marker for ccRCC patients and may play a key role in tumor microenvironment modulation.

## GRAPHICAL ABSTRACT

**Figure:**
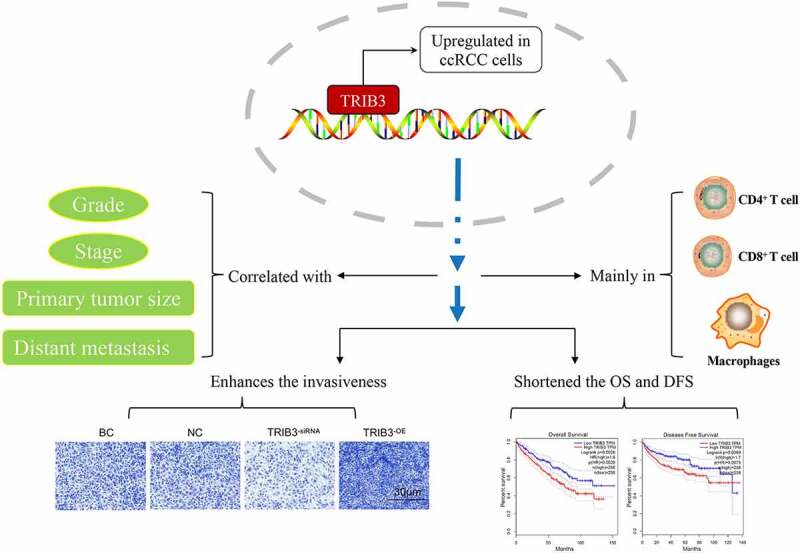

## Highlights

- Elevated TRIB3 levels are associated with poorer clinicopathological variables
- TRIB3 overexpression enhances the invasiveness of ccRCC cells
- High TRIB3 levels represent an independent risk factor for OS
- Localization and binding targets of TRIB3 and 361 DEGs related to it are identified

## 1. Introduction

Renal cell carcinoma (RCC) is one of the most common tumors of the urinary system [1]. Worldwide, approximately 400,000 people are diagnosed with RCC and 175,000 die from the disease annually [2], and its incidence continues to rise. RCC demonstrates histological subtypes, the most common being kidney renal clear cell carcinoma (ccRCC) [3]. Recently, the incidence of ccRCC has increased [4]. The identification of molecular markers with high specificity for ccRCC is crucial for effective early diagnosis, treatment, and accurate prediction of prognosis, and it is also important for the designing individualized treatment regimens.

TRIB3 is the mammalian homolog of the *Drosophila Trbl* gene [5] which encodes a pseudokinase that acts as a negative regulator of various signaling pathways. Early studies showed that TRIB3 plays an important role in apoptosis, differentiation, and the cellular responses to stress [6–8]. Recent studies have found that *TRIB3* is highly expressed in many tumors, including breast [9], lung [10,11], colorectal [12], and liver [13] cancers, and it is closely related to tumor stage, recurrence, and prognosis [14]. *TRIB3* has also been shown to plays an anti-apoptotic role in doxorubicin-treated gastric cancer cell lines and be highly expressed in gastric cancer tumors, and is related to poor patient prognosis [15]. In addition, high *TRIB3* expression correlates with advanced clinical stage and poor differentiation in many cancers [16]. Several studies have also shown that *TRIB3* knockdown inhibits the growth of rectal cancer and breast cancer cells [17,18]. Collectively, these results suggest a role for TRIB3 in promoting cancer growth and indicate that TRIB3 may be a prognostic marker and/or therapeutic target. However, the role of TRIB3 in ccRCC or its correlation with clinicopathological features and prognosis remains unknown.

Thus, it is suggested that TRIB3 may be a safe and effective novel target for tumor therapy. TRIB3 may be highly expressed in ccRCC, which preliminarily unveils the role of TRIB3 in the occurrence and development of RCC, but the relationship and mechanism involving TRIB3 and clinicopathological variables in ccRCC remain unclear; hence, further research is needed. In this study, we explored the correlation between TRIB3 expression and clinical pathological grade in ccRCC and the prognosis of patients in order to clarify the influence of promoting or inhibiting TRIB3 expression on cell biological behavior, such as the invasiveness of ccRCC cells. Concurrently, we explored DEGs related to TRIB3 expression and potential signaling pathways involving TRIB3 and clarified the correlation between TRIB3 and tumor-infiltrating immune cells in ccRCC. This is of great significance to further explore the molecular regulatory mechanisms in ccRCC and can provide novel therapeutic strategies and targets for developing new methods for the diagnosis and treatment of ccRCC.

## 2. Materials and methods

### 2.1. RNA sequencing datasets and bioinformatics analysis

The ccRCC gene expression dataset (611 samples, Workflow Type: HTSeq-FPKM) and corresponding clinical information were downloaded from The Cancer Genome Atlas (TCGA) in June 2021. Of the 611 samples, 72 were normal paracancerous kidney samples and 539 were ccRCC samples. After excluding 47 samples with incomplete information, the dataset consisted of 72 normal samples and 496 ccRCC samples (Table 1). The Gene Expression Profiling Interactive Analysis (GEPIA) portal was also employed. The GEPIA ccRCC dataset consists of 100 normal samples and 523 ccRCC samples. For analysis of TRIB3-high and TRIB3-low groups within TCGA dataset, patients were assigned to two groups using the median TRIB3 expression level as the cutoff value.

**Table 1.** Clinicopathological characteristics of patients in TCGA ccRCC dataset.

Clinical characteristics		Total (496)	%
Age at diagnosis (y)	<60 ≥60	228 268	45.97 54.03
Grade	G1	10	2.02
	G2	214	43.15
	G3	196	39.52
	G4	76	15.32
Stage	I	242	48.79
	II	51	10.28
	III	122	24.60
	IV	81	16.33
Gender	Female	171	34.48
	Male	325	65.52
Tumor size	T1	248	50.00
	T2	62	12.50
	T3	175	35.28
	T4	11	2.22
Node	NX	248	50.00
	N0	233	46.98
	N1	15	3.02
Metastasis	M0	418	84.27
	M1	78	15.73

### 2.2 Identification of DEGs related to TRIB3 expression and functional enrichment analysis

The DEGs between low and high *TRIB3* expression groups (cutoff value was set as median expression level of *TRIB3*) were identified using the limma package [19]. DEGs with log|FC| (fold change) ≥ and P-value <0.01 were considered statistically significant. Then the DEGs were uploaded to the Search Tool for the Retrieval of Interacting Genes/Proteins (STRING; http://string-db.org) and a protein–protein interaction (PPI) network was constructed (interaction score was set > 0.900). Next, functional enrichment analysis based on gene ontology (GO) [20] and Kyoto Encyclopedia of Genes and Genomes (KEGG) [21] databases were utilized to explore the potential biological functions of the DEGs by using the ClusterProfiler package [22].

### 2.3. Abundance of tumor-infiltrating lymphocytes (TILs) and the single-cell sequencing across ccRCC

The Tumor IMmune Estimation Resource algorithm database (https://cistrome.shinyapps.io/timer/) was used to estimate the abundance of TILs in the ccRCC samples [23]. Meanwhile, the single-cell RNA-seq datasets, GSE111360, GSE139555, and GSE145281, were investigated in this study from the Tumor Immune Single-cell Hub to characterize tumor microenvironments at the single-cell resolution [24].

### 2.4. Gene set enrichment analysis (GSEA)

GSEA was performed using the GSEA 4.1.0 software (http://software.broadinstitute.org/gsea/index.jsp) [25]. KEGG pathway enrichment analysis was performed using the dataset release number v.7.4. The TRIB3-high and TRIB3-low expression groups were used as phenotype labels. Enrichment was performed 1,000 times, and the nominal P-value and normalized enrichment scores were used to identify pathways enriched for each phenotype.

### 2.5. TRIB3 association with immune phenotype

The Tumor and Immune System Interaction Database (TISIDB; http://cis.hku.hk/TISIDB/index.php) was used to analyze the relationship between TRIB3 and tumor immunity [26]. TISIDB is a web portal that integrates multiple heterogeneous data types. We screened all tumor immune factors related to TRIB3 using the parameters (i) relationships between abundance of 28 TIL subtypes and expression, copy number, methylation, or mutation of TRIB3, (ii) relationships between three kinds of immunomodulators and the expression, copy number, methylation, or mutation of *TRIB3*, and (iii) the distribution of *TRIB3* expression across immune and molecular subtypes, and selected for presentation those most significantly related to *TRIB3*.

### 2.6. Cell lines, culture conditions, and reagents

786O cells were obtained from ATCC, and was maintained in DMEM (Gibco, 11960044) supplemented with 10% fetal bovine serum (Gibco). Transwell assay-related reagent consumables were as follows: Matrigel Basement Membrane Matrix (BD, #356234, USA), Costar 6.5-mm Transwell Permeable Support with 8.0-μm Pore Polycarbonate Membrane (Corning, #3422, USA), Crystal Violet Stain solution, 0.1% (Solarbio, #G1063, China). TRIB3-overexpression plasmid (Qingke, China). TRIB3 target sequences: Human-TRIB3-siRNA-F: 5′-GGAGUUGGAUGACAACUUATT-3′; Human-TRIB3-siRNA-R: 5′-UAAGUUGUCAUCCAACUCCTT-3′.

### 2.7. Reverse transcription and quantitative reverse transcription-PCR (RT-qPCR)

Total RNA was isolated from cultured cells and converted into cDNA using specific primers and the HiScript III cDNA synthesis kit (Vazyme, Nanjing, China). The following primers were used: TRIB3-F: 5′-CGAGGCCGTCACCAAGAAC-3′, TRIB3-R: 5′-GTAGTGGTCGATGCGGTAGA-3′. The mRNA levels of IR were determined using RT-qPCR on a CFX96 Touch real-time PCR detection system (Bio-Rad, Hercules, CA, USA). Actin was used as an internal reference gene.

### 2.8. Western blot analysis

Cultured cells were lysed using 0.5% NP-40 buffer containing 50 mM Tris-HCl (pH 7.5), 150 mM NaCl, 0.5% Nonidet P-40, and a cocktail of protease inhibitors (Sigma-Aldrich, St. Louis, Missouri, USA). After centrifugation at 16,000 × g at 4°C for 15 min, lysate supernatants were analyzed using western blotting according to standard procedures. Anti-TRIB3 antibody (ABclonal, A5424, 1:1000). Protein abundance was detected by measuring chemiluminescence on a Typhoon FLA 9500 instrument (GE Healthcare, Little Chalfont, UK).

### 2.9. Statistical analyses

All statistical analyses were performed using the R software (v.3.6.3). Wilcoxon’s signed rank test and logistic regression were used to analyze the relationship between clinicopathological characteristics and TRIB3 levels. Cox regression and Kaplan–Meier methods were used to analyze clinicopathological features associated with OS. Multivariate Cox analysis was used to compare the relationships among TRIB3 expression, survival, and other clinical features. Spearman’s correlation coefficient was used to examine relationships between TRIB3 and immune parameters. P < 0.05 was considered statistically significant.

## 3. Result

Our study was the first to systematically reveal the correlation between TRIB3 and clinical pathological grade of ccRCC and patient prognosis and to clarify the influence of promoting or inhibiting TRIB3 in terms of cell biological behaviors such as the invasiveness of ccRCC cells. Simultaneously, we explored the DEGs related to TRIB3 expression and potential signaling pathways related to TRIB3 and clarified the correlation between TRIB3 and tumor-infiltrating immune cells in ccRCC, which is novel and of great significance.

### 3.1. Patient characteristics

Clinicopathological and gene expression data for 496 patients with confirmed primary ccRCC were downloaded from TCGA (Table 1). Of the 496 patients, 325 (65.52%) were male and 268 (54.03%) were aged above 60 years. In our cohort, tumor grade frequencies were G1, 10 cases (2.02%); G2, 214 cases (43.15%); G3, 196 cases (39.52%); and G4, 76 cases (15.32%). There were 242 cases (48.79%) with stage I disease, 51 cases (10.28%) with stage II, 122 cases (24.60%) with stage III, and 81 (16.33%) stage IV cases. With respect to tumor–node–metastasis (TNM) staging, most cases were T1, N0, M0, and distant metastasis was absent in 418 patients (84.27%).

### 3.2. Expression of TRIB3 in ccRCC and adjacent tissues

**Figure 1.**
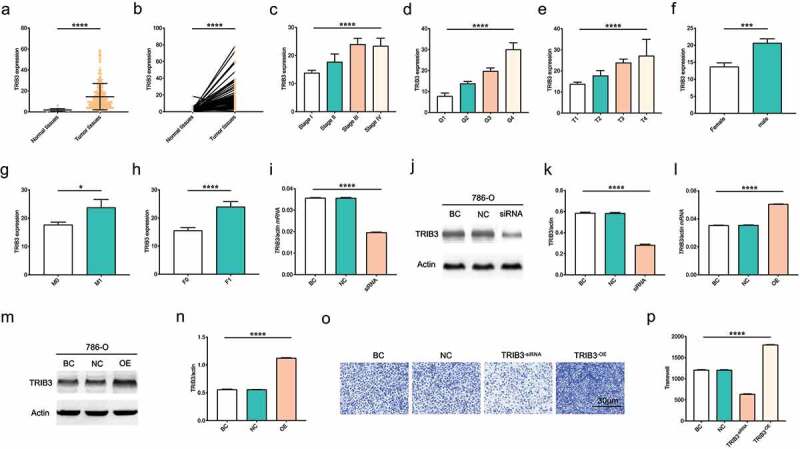
Associations between TRIB3 expression and clinicopathologic characteristics of ccRCC patients.

We first analyzed the expression of TRIB3 in 496 ccRCC tissues and 72 adjacent normal tissues in TCGA dataset. Notably, TRIB3 was expressed at significantly higher levels in tumor tissue than in normal kidney tissue (Figure 1(a), p < 0.001). Similar results were obtained for the evaluation of TRIB3 in 72 pairs of tumor samples and paracancerous tissues in TCGA dataset, which showed that TRIB3 was expressed at significantly higher levels in ccRCC tissue than in normal kidney tissue (Figure 1(b), p < 0.001). These results suggested that TRIB3 expression was dysregulated in ccRCC; thus, it may play an important role in promoting tumor growth.

(a) Expression of TRIB3 in ccRCC and adjacent normal kidney tissues in TCGA dataset. (b) Expression of TRIB3 in ccRCC and matched paracancerous normal tissues in TCGA dataset. (c-h) Wilcoxon’s and Kruskal tests showing associations between TRIB3 expression levels and (c) stage, (d) grade, (e) gender, (f) primary tumor, (g) distant metastases, and (h) fustat of patient. (i) Transfection efficiency of TRIB3-siRNA was detected using RT-qPCR. (j-k) Transfection efficiency of TRIB3-siRNA was detected using western blotting. (l) Transfection efficiency of TRIB3-overexpression plasmid was detected using RT-qPCR. (m, n) Transfection efficiency of TRIB3-overexpression plasmid was detected using western blotting. (o) Transwell assays detected the effects of inhibiting TRIB3 expression and overexpression of TRIB3 on cell invasiveness.

### 3.3. Associations between TRIB3 expression in ccRCC and clinicopathological variables

We next determined whether TRIB3 expression in ccRCC tumors correlated with common clinicopathological variables. The 496 patients in TCGA dataset were assigned to high and low TRIB3 expression groups using the median TRIB3 expression level as the cutoff. As shown in Figure 1(c–h), high TRIB3 expression was significantly correlated with clinical stage (P < 0.001), pathological grade (P < 0.001), gender (P < 0.001), primary tumor size (P < 0.001), distant metastasis (P = 0.003), and patient follow-up status (fustat). Univariate Cox regression analysis showed that high TRIB3 levels were significantly associated with poor prognosis (Table 2); that is, high TRIB3 expression correlated significantly with grade (hazard ratio [HR] = 7.37 for G4 vs G1), stage (HR = 1.87 for stage III vs stage I, HR = 2.13 for stage IV vs stage I), primary tumor (HR = 2.05 for T3 vs T1), distant metastasis (HR = 1.98 for M1 vs M0), and gender (HR = 2.17 for male vs female) (all P < 0.05).

**Table 2.** Logistic regression analysis of the associations between TRIB3 expression and clinicopathological characteristics.

Clinical characteristics	Total (N)	Hazard ratio in TRIB3 expression	p-Value
Grade (G4 vs. G1)	89	7.37 (2.19–29.48)	.**002**
Stage (III vs. I)	388	1.87 (1.21–2.89)	.**004**
Stage (IV vs. I)	347	2.13 (1.29–3.57)	.**003**
Tumor size (T3 vs. T1)	450	2.05 (1.40–3.01)	.**000**
Metastasis (M1 vs. M0)	496	1.98 (1.21–3.31)	.**007**
Gender (male vs. female)	496	2.17 (1.51–3.13)	.**000**

### 3.4. Effects of inhibiting TRIB3 expression and overexpression of TRIB3 on cell invasiveness as seen using Transwell assays

To examine the functional role of TRIB3 in ccRCC in vitro, we constructed TRIB3-siRNA and TRIB3-overexpression plasmids to study the effects of TRIB3 on the biological behavior of ccRCC cells, and we selected the invasive force of cells as the measurement index. We verified the transfection efficiency of TRIB3-siRNA and TRIB3-overexpression plasmids using RT-qPCR and western blotting, respectively (Figure 1(i–n)). Results showed that inhibiting the expression of TRIB3 subdued the invasiveness of ccRCC cells, whereas promoting the expression of TRIB3 enhanced the invasiveness of ccRCC cells (Figure 1(o,p)). Therefore, the upregulation of TRIB3 expression is related to advanced disease and distant metastasis and positively correlated with the invasiveness of ccRCC cells.

### 3.5. Survival outcomes and Cox regression analysis

**Figure 2.**
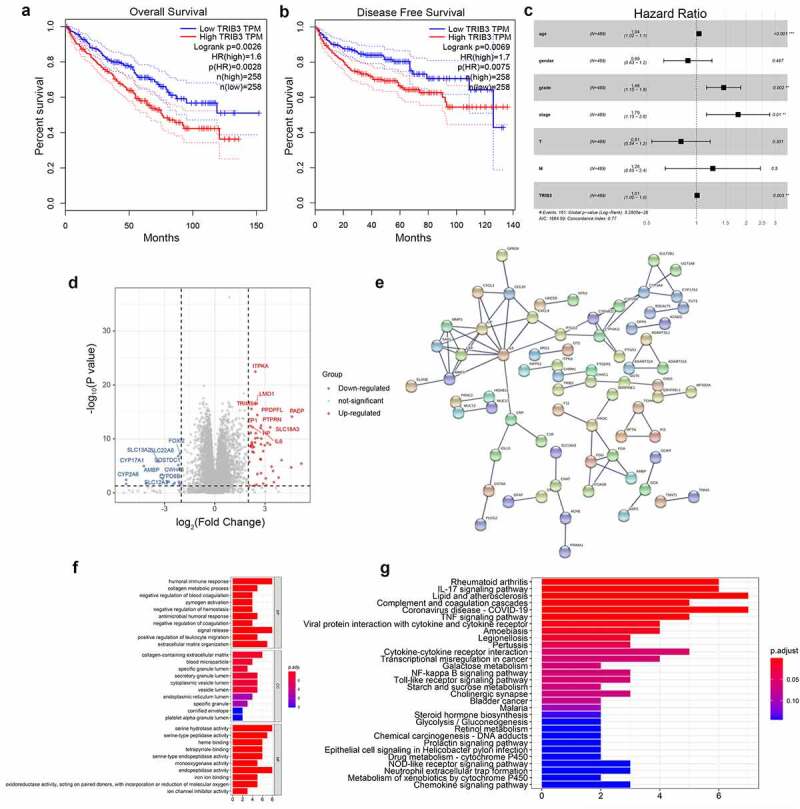
Survival outcomes and Cox regression analysis in ccRCC patients.

Kaplan–Meier survival analysis of TCGA ccRCC dataset showed that patients with high TRIB3-expressing tumors had significantly shorter survival times than patients with low TRIB3-expressing tumors (Figure 2(a), p < 0.001). The results of this analysis was verified using an independent GEPIA dataset (Figure 2(b), p < 0.001). Univariate analysis of clinicopathological characteristics and OS of the patients in TCGA ccRCC cohort showed that age, grade, stage, T, M, and TRIB3 expression were significantly correlated with OS (P < 0.001; Table 3), and age, grade, stage, and TRIB3 expression remained significantly associated with OS in multivariate analysis (Figure 2(c), p < 0.05). Other factors, such as lymph node infiltration, were not included in the analysis because of the large number of Nx cases.

**Table 3.** Univariate and multivariate analysis of the associations between TRIB3 expression and overall survival of ccRCC patients.

Parameter	Univariate analysis			Multivariate analysis		
	HR	95% CI	p-Value	HR	95% CI	p-Value
Age	1.03	1.02–1.05	**0.000**	1.04	1.02–1.05	.**000**
Grader	2.29	1.85–2.84	**0.000**	1.46	1.15–1.85	.**002**
Stage	1.88	1.65–2.16	**0.000**	1.79	1.15–2.78	.**010**
Tumor size	1.94	1.63–2.30	**0.000**	0.81	0.54–1.21	.301
Metastasis	4.28	3.11–5.91	**0.000**	1.26	0.65–2.44	.500
TRIB3	1.01	1.01–1.02	**0.000**	1.01	1.00–1.02	.**003**

Bold values indicate P < 0.05. HR, hazard ratio; CI, confidence interval.

(a) Kaplan–Meier OS analysis of ccRCC patients stratified by tumor expression of TRIB3. (b) Kaplan–Meier disease-free survival analysis of ccRCC patients stratified by tumor expression of TRIB3. (c) Forest map showing correlations between OS and TRIB3 expression, age, grade, and stage. (d, e) Volcano map and PPI network of DEGs. (f) Functional enrichment analysis of DEGs based on the GO database. (g) Functional enrichment analysis of DEGs based on the KEGG database.

### 3.6. DEGs related to TRIB3 expression were identified

As depicted in Figure 2(d), a total of 361 DEGs related to TRIB3 were identified and DEGs with most significant changes included ITPKA, LMO1, TRIM54, and PPDPFL. A PPI network was constructed using STRING (Figure 2(e)). Functional enrichment analysis (Figure 2(f,g), Table 4) indicated that the DEGs were mostly enriched in humoral immune responses, collagen-containing extracellular matrix, serine hydrolase activity and rheumatoid arthritis.

**Table 4.** GO (a) and KEGG (b) pathways enrichment analysis of DEGs in the most significant module.

Term	Description	Gene Ratio	p-Value (adjusted)
a.			
GO:0017171	serine hydrolase activity	8/42	1.87E-06
GO:0020037	heme binding	6/42	4.13E-05
GO:0004497	monooxygenase activity	5/42	0.000100021
GO:0006959	humoral immune response	8/43	0.001652096
GO:0023061	signal release	8/43	0.001652096
GO:0050714 GO:0062023	positive regulation of protein secretion	6/43 6/43	0.0029326340.012025886 0.012025886
GO:0072562	collagen-containing extracellular	4/43	0.012025886
GO:0035580	matrix	3/43	
b.	blood microparticle		7.54E-05
hsa04657	specific granule lumen	6/31	0.001220886
hsa04668	IL-17 signaling pathway	5/31	0.008917891
hsa04061	TNF signaling pathway Viral protein interaction with cytokine and cytokine receptor	4/31	

Abbreviations: GO: Gene Ontology; KEGG: Kyoto Encyclopedia of Genes and Genomes; DEGs: differentially expressed genes.

### 3.7. Abundance of TILs and single-cell sequencing across ccRCC

**Figure 3.**
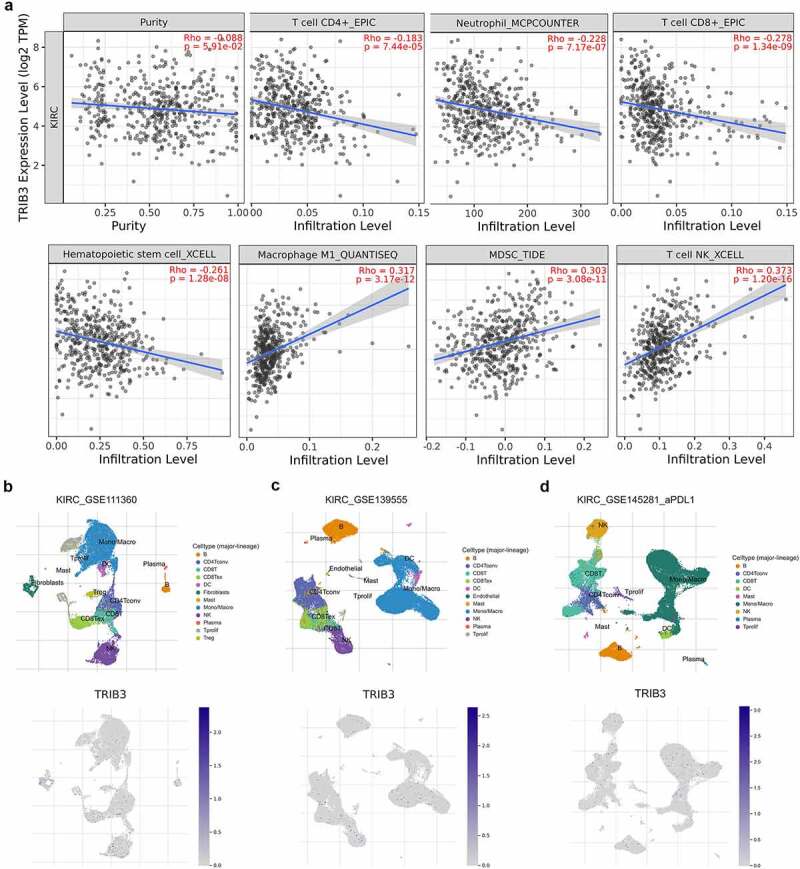
Abundance of tumor-infiltrating lymphocytes (TILs) and single-cell sequencing across ccRCC.

As shown in Figure 3(a), TRIB3 expression exhibited a significantly negative association with CD8^+^ T (cor. = −0.278) and hematopoietic stem cell (cor. = −0.261) numbers and positive association with NK T cells (cor. = 0.373) and macrophage M1 (cor. = 0.317). As shown in Figure 3(b–d), the single-cell sequencing dataset GSE111360, GSE139555, and GSE145281 suggested localization and binding targets of TRIB3 mainly in monocytes/macrophages and CD4^+^ and CD8^+^ T cells.

(a) TRIB3 expression was significantly negatively associated with CD8^+^ T (cor. = −0.278) and hematopoietic stem cell counts (cor. = −0.261) and positively associated with NK T (cor. = 0.373) and macrophage M1 cell counts (cor. = 0.317). (b-d) Single-cell sequencing datasets GSE111360, GSE139555, and GSE145281 suggested localization and binding targets of TRIB3 mainly involve monocytes/macrophages and CD4^+^ and CD8^+^ T cells.

### 3.8. GSEA identifies potential TRIB3-related signaling pathways

**Figure 4.**
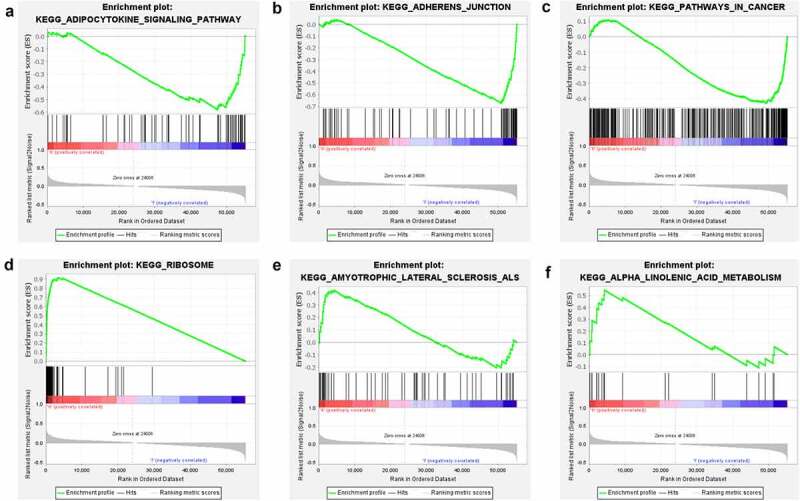
Gene set enrichment analysis of TRIB3 expression in ccRCC.

To investigate the involvement of TRIB3 in ccRCC, we performed GSEA for tumors with high and low TRIB3 expression. GSEA showed that TRIB3 was enriched in distinct pathways for the two tumor phenotypes. Thus, ‘adipocytokine signaling pathway’, ‘adherens junction’, and ‘pathways in cancer’ were enriched in the TRIB3-low expression phenotype, whereas ‘ribosome’, ‘amyotrophic lateral sclerosis’, and ‘alpha linolenic acid metabolism’ were enriched in the TRIB3-high expression phenotype (Figure 4(a–f)).

GSEA results showing differential pathway enrichment of TRIB3 expression in tumors with the TRIB3-low phenotype – (a) adipocytokine signaling pathway, (b) adherens junction, (c) pathways in cancer – and tumors with the TRIB3-high phenotype – (d) ribosome, (e) amyotrophic lateral sclerosis, and (f) alpha linolenic acid metabolism.

### 3.9. TRIB3 correlations with tumor-infiltrating immune cells in ccRCC

**Figure 5.**
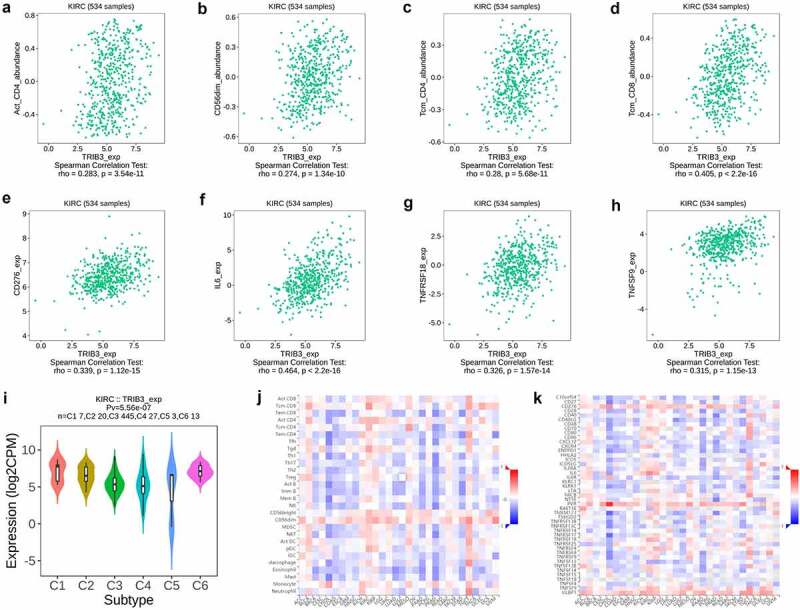
TRIB3 expression differentially correlates with immune cell infiltration in ccRCC and other cancers.

To explore the relationship between TRIB3 expression and tumor immunity, we performed Spearman’s correlation analysis in TISIDB using the immunostimulator (which samples a range of immunostimulatory molecules) and lymphocyte settings (which samples the abundance of TILs across a range of human cancers). We found that the expression of TRIB3 was positively correlated with the expression of immunostimulator and abundance of TILs in ccRCC (Figure 5(j,k)), and was significantly correlated with the abundance of activated CD4^+^ T cells (r = 0.283, Figure 5(a)), CD56dim natural killer cells (r = 0.274, Figure 5(b)), central memory CD4^+^ T cells (r = 0.28, Figure 5(c)), and central memory CD8^+^ T cells (r = 0.405, Figure 5(d)), as well as with increased expression of CD276 (r = 0.339, Figure 5e), IL-6 (r = 0.464, figure 5(f)), TNFRSF18 (r = 0.326, Figure 5(g)), and TNFSF9 (r = 0.315, Figure 5(h)). Finally, Figure 5(i) shows that TRIB3 expression was widely distributed in different immune subtype – wound healing (C1), IFN-gamma dominant (C2), inflammatory (C3), lymphocyte-depleted (C4), immunologically quiet (C5), and TGF-b dominant (C6) – in ccRCC, indicating that TRIB3 has influence on all immune phenotypes.

T RIB3 expression analysis and correlation identification were performed using TISIDB. (a–d) Spearman’s correlation analysis between TRIB3 expression and activated CD4^+^ T (r = 0.283), CD56dim natural killer (r = 0.274), central memory CD4^+^ T (r = 0.28), and central memory CD8^+^ T cells (r = 0.405). (e–h) Spearman’s correlation analysis between TRIB3 expression and CD276 (r = 0.339), IL-6 (r = 0.464), TNFRSF18 (r = 0.326), and TNFSF9 (r = 0.315) expression. (i) TRIB3 expression in ccRCC immune cell subsets in ccRCC. (j, k) Spearman’s correlation analysis between TRIB3 expression, immunostimulatory molecules, and abundance of tumor-infiltrating lymphocytes (TILs) in multiple cancers (j) and in ccRCC (k).

## 4. Discussion

ccRCC is a malignant tumor that has devastating effects on human health. Moreover, the economic burden of ccRCC is substantial, affecting both families and society. It is important to have accurate and concise information about the pathology of ccRCC when treating patients. This will improve the decision-making process related to patient care and individualized treatments; therefore, it is important to use various approaches, such as data mining, to find robust molecular markers and therapeutic targets to use in the diagnosis, prognosis, and treatment of ccRCC.

As a stress sensor, TRIB3 responds to a variety of stresses [27–29]. It regulates homeostasis, metabolic diseases, and cancer by interacting with intracellular signals and functional protein pathways [5,30,31]. In this study, we implemented computational biology methods and experimental verification techniques to identify and characterize the role of TRIB3 in the development, progression, and metastasis of ccRCC. We demonstrated the efficiency of integrating bioinformatics with in vitro experiments to explore potential biomarkers and therapeutic targets for ccRCC. Furthermore, we revealed the role of TRIB3 in the malignant progression, immune landscape characteristics, tumor microenvironment, and clinical outcomes of patients with ccRCC. These findings encourage further exploration of the pathogenesis of ccRCC.

Previous studies have shown that TRIB3 expression is elevated in several types of cancer, and it promotes the migration of tumor cells through modulation of various oncoproteins [11,12,32,33]. Inhibiting the expression of TRIB3 may significantly reduce the occurrence and progression of cancer [34,35]. In this study, we found that the expression of TRIB3 increased with the advancement of clinical stage, pathological grade, primary tumor size, distant metastasis, and patient fustat, suggesting that TRIB3 promotes ccRCC progression and increases the risk of invasion and deterioration of ccRCC. Kaplan–Meier analysis showed that high expression of TRIB3 correlated with shorter OS rates. These results suggest that the expression level of TRIB3 may serve as an index to predict clinical outcomes in patients with ccRCC. Similarly, in lung, breast, and ovarian cancers, high TRIB3 expression promotes tumor malignancy [9,11,36]. Evidently, TRIB3 may play a tumor-promoting role.

In addition, TRIB3 is closely associated with tumor immunity. The immune landscape characteristics examined in this study showed that TRIB3 expression had a significant negative association with CD8^+^ T and hematopoietic stem cells; by contrast, it had a positive association with NK cells and M1 macrophages. Single-cell sequencing revealed the binding targets of TRIB3 and their locations, which were mainly in monocytes/macrophages, CD4^+^ T cells, and CD8^+^ T cells. The association of TRIB3 and tumor immunity has been reported in other types of tumors [37,38]; for example, in colorectal cancer, TRIB3 can reduce CD8^+^ T cell infiltration and induce immune evasion by inhibiting the STAT1-CXCL10 axis, suggesting that TRIB3 may be an attractive therapeutic target for ‘warming up’ of immune-resistant ‘cold’ tumors [37].

The mechanism of TRIB3 has been investigated; previous studies have shown that TRIB3 promotes MYC-associated lymphoma development through suppression of UBE3B-mediated MYC degradation [33]. Additionally, TRIB3 interacts with β-catenin and TCF4 to increase the stem cell features of colorectal cancer stem cells and trigger tumorigenesis [12]. In our study, we performed enrichment analysis of 361 DEGs related to TRIB3. The analysis showed that TRIB3 was related to humoral immune responses, the collagen-containing extracellular matrix, serine hydrolase activity, and rheumatoid arthritis. This highlights the focus areas for future research.

Thus, we concluded that TRIB3 is a potential oncogene and therapeutic target for ccRCC.

## 5. Conclusion

In summary, this study provided the first evidence demonstrating that TRIB3 knockdown or overexpression can affect cell invasiveness and revealed that high levels of TRIB3 represent an independent risk factor for OS. Immune landscape characterization showed that TRIB3 was significantly associated with the tumor immune microenvironment in ccRCC, including altered patterns of TILs. Elevated TRIB3 expression is a promising prognostic marker for ccRCC patients and may play a key role in tumor microenvironments, which indicates a novel molecular mechanism involving TRIB3 in ccRCC and identifies TRIB3 as a novel therapeutic target for ccRCC therapy from bench to clinic.

## Supplementary Material

Supplemental Material
