# Supplementary material for: Increased expression of tribbles homolog 3 predicts poor prognosis and correlates with tumor immunity in clear cell renal cell carcinoma: a bioinformatics study
Source: Bioengineered. 2022 Jun 20;13(5):14000–12. doi: 10.1080/21655979.2022.2086380 (PMC9275882; doi:10.1080/21655979.2022.2086380)
Supplement: Supplemental Material [file KBIE_A_2086380_SM0173.zip › supplementary/Supplementary response.docx]

1. Please include a statement in the methods section of your article indicating what ethical approval was obtained for the study.

**Response:**

Thank you for your helpful suggestions. Ethical approval is not required for this study for the following reasons.

1. The ccRCC gene expression dataset (611 samples, Workflow Type: HTSeq-FPKM) and corresponding clinical information were downloaded from The Cancer Genome Atlas (TCGA) in June 2021.
2. The DEGs were uploaded to the Search Tool for the Retrieval of Interacting Genes/Proteins (STRING; http://string-db.org) and a protein–protein interaction (PPI) network was constructed (interaction score was set > 0.900).
3. The Tumor IMmune Estimation Resource algorithm database (https://cistrome.shinyapps.io/timer/) was used to estimate the abundance of TILs in the ccRCC samples.
4. The single-cell RNA-seq datasets, GSE111360, GSE139555, and GSE145281, were investigated in this study from the Tumour Immune Single-cell Hub to characterize tumor microenvironments at the single-cell resolution.
5. The Tumor and Immune System Interaction Database (TISIDB; http://cis.hku.hk/TISIDB/index.php) was used to analyze the relationship between TRIB3 and tumor immunity.
6. The 786O cells were obtained from ATCC.

We have cited references for all the data download and research methods mentioned above.

2. Please provide a declaration of funding at the end of your article before the references.

**Response:**

Thank you for your valued suggestions. We have supplemented the funding declaration, lines 364–369, as shown by the different font color.
