# Supplementary figures and images for: Increased expression of tribbles homolog 3 predicts poor prognosis and correlates with tumor immunity in clear cell renal cell carcinoma: a bioinformatics study
Source: Bioengineered. 2022 Jun 20;13(5):14000–12. doi: 10.1080/21655979.2022.2086380 (PMC9275882; doi:10.1080/21655979.2022.2086380)

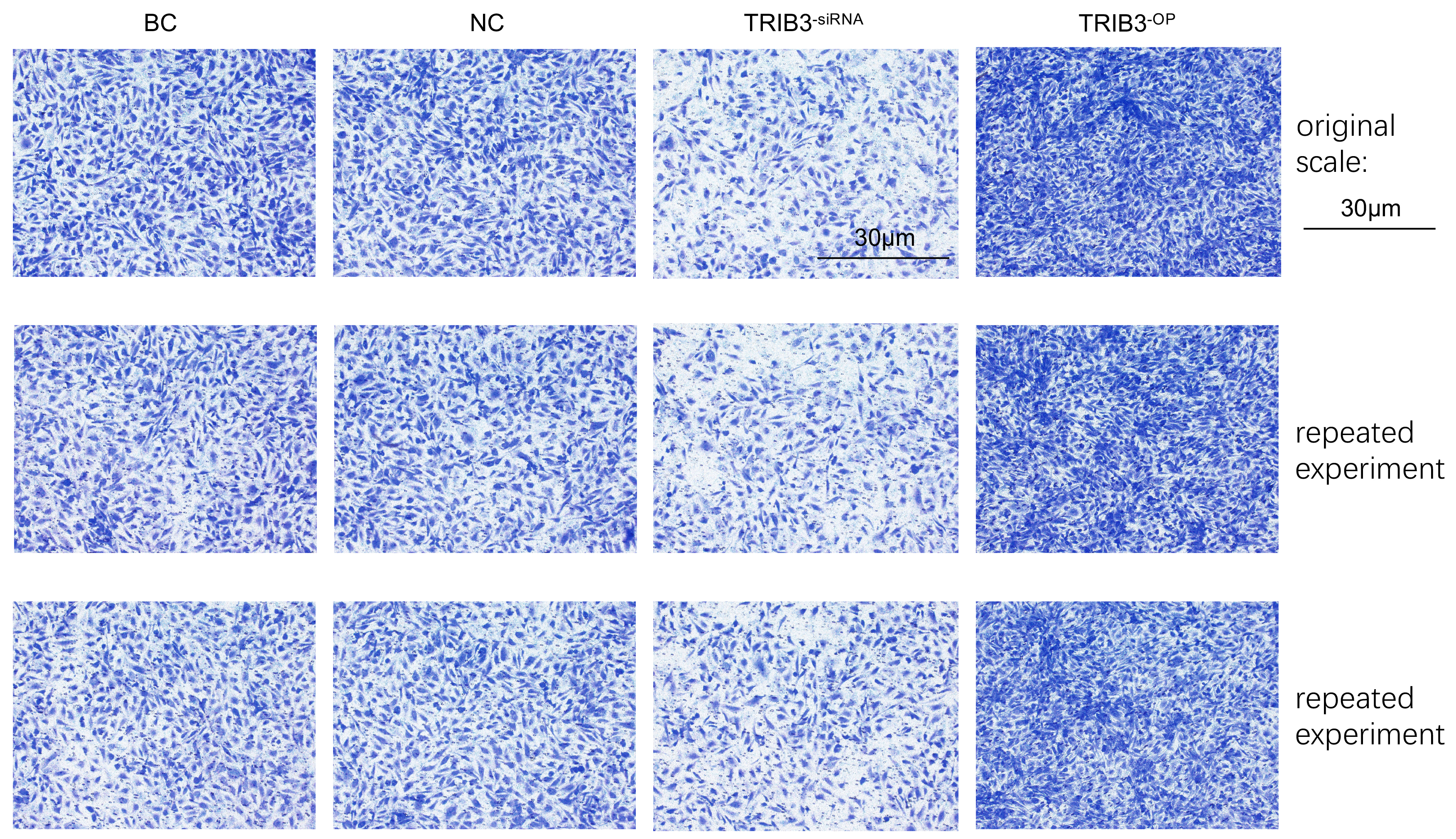

Supplement: Supplemental Material [file KBIE_A_2086380_SM0173.zip › supplementary/microscopy images.jpg]

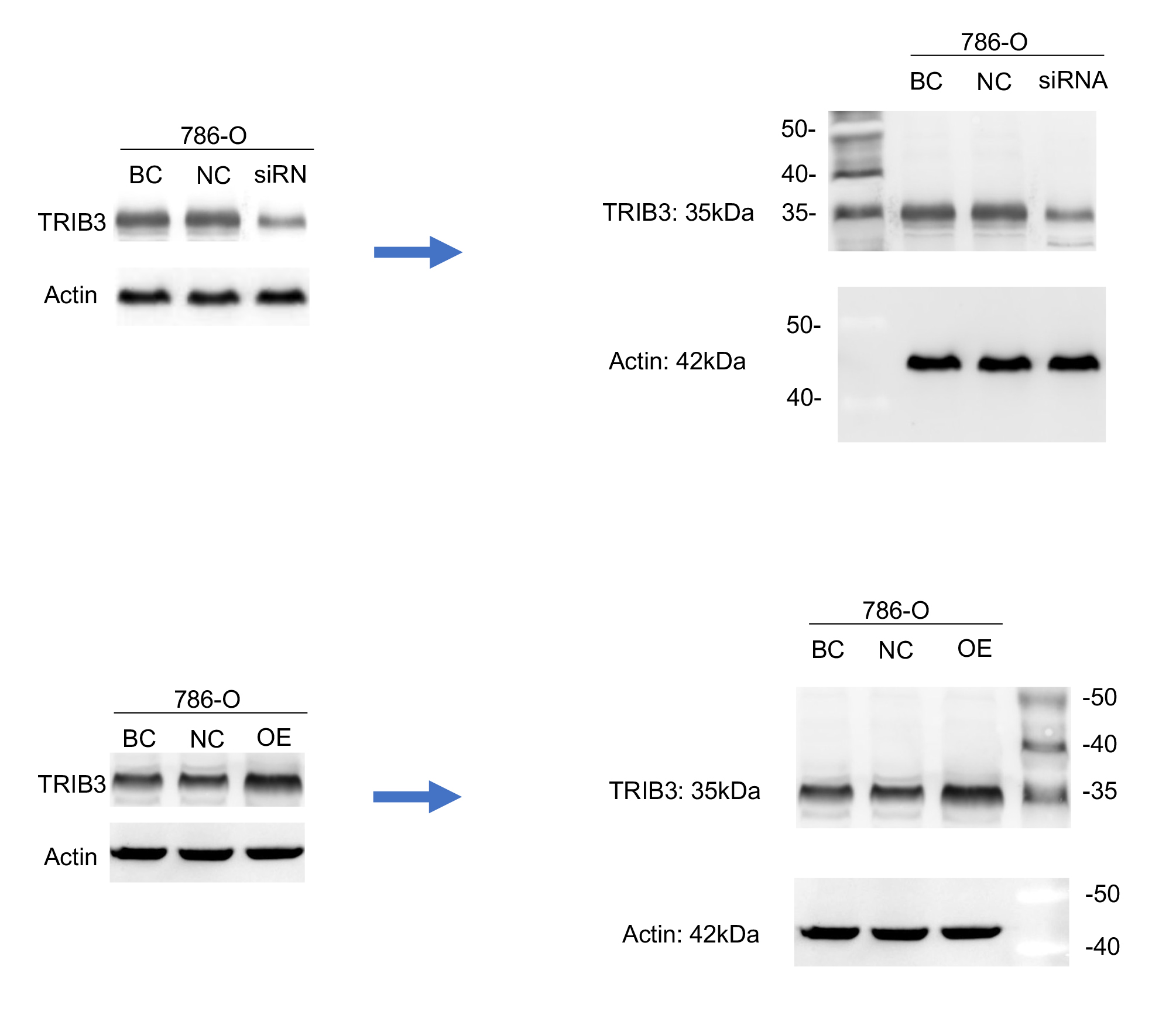

Supplement: Supplemental Material [file KBIE_A_2086380_SM0173.zip › supplementary/original images of western blot.jpg]
